# A detailed 3D MRI brain atlas of the African lungfish *Protopterus annectens*

**DOI:** 10.1038/s41598-024-58671-x

**Published:** 2024-04-05

**Authors:** Daniel Lozano, Jesús M. López, Adrián Chinarro, Ruth Morona, Nerea Moreno

**Affiliations:** Department of Cell Biology, Faculty of Biological Sciences, Complutense University, 28040 Madrid, Spain

**Keywords:** 3-D reconstruction, Magnetic resonance imaging, Neuroscience

## Abstract

The study of the brain by magnetic resonance imaging (MRI) in evolutionary analyses is still in its incipient stage, however, it is particularly useful as it allows us to analyze detailed anatomical images and compare brains of rare or otherwise inaccessible species, evolutionarily contextualizing possible differences, while at the same time being non-invasive. A good example is the lungfishes, sarcopterygians that are the closest living relatives of tetrapods and thus have an interesting phylogenetic position in the evolutionary conquest of the terrestrial environment. In the present study, we have developed a three-dimensional representation of the brain of the lungfish *Protopterus annectens* together with a rostrocaudal anatomical atlas. This methodological approach provides a clear delineation of the major brain subdivisions of this model and allows to measure both brain and ventricular volumes. Our results confirm that lungfish show neuroanatomical patterns reminiscent of those of extant basal sarcopterygians, with an evaginated telencephalon, and distinctive characters like a small optic tectum. These and additional characters uncover lungfish as a remarkable model to understand the origins of tetrapod diversity, indicating that their brain may contain significant clues to the characters of the brain of ancestral tetrapods.

## Introduction

**Figure 1:**
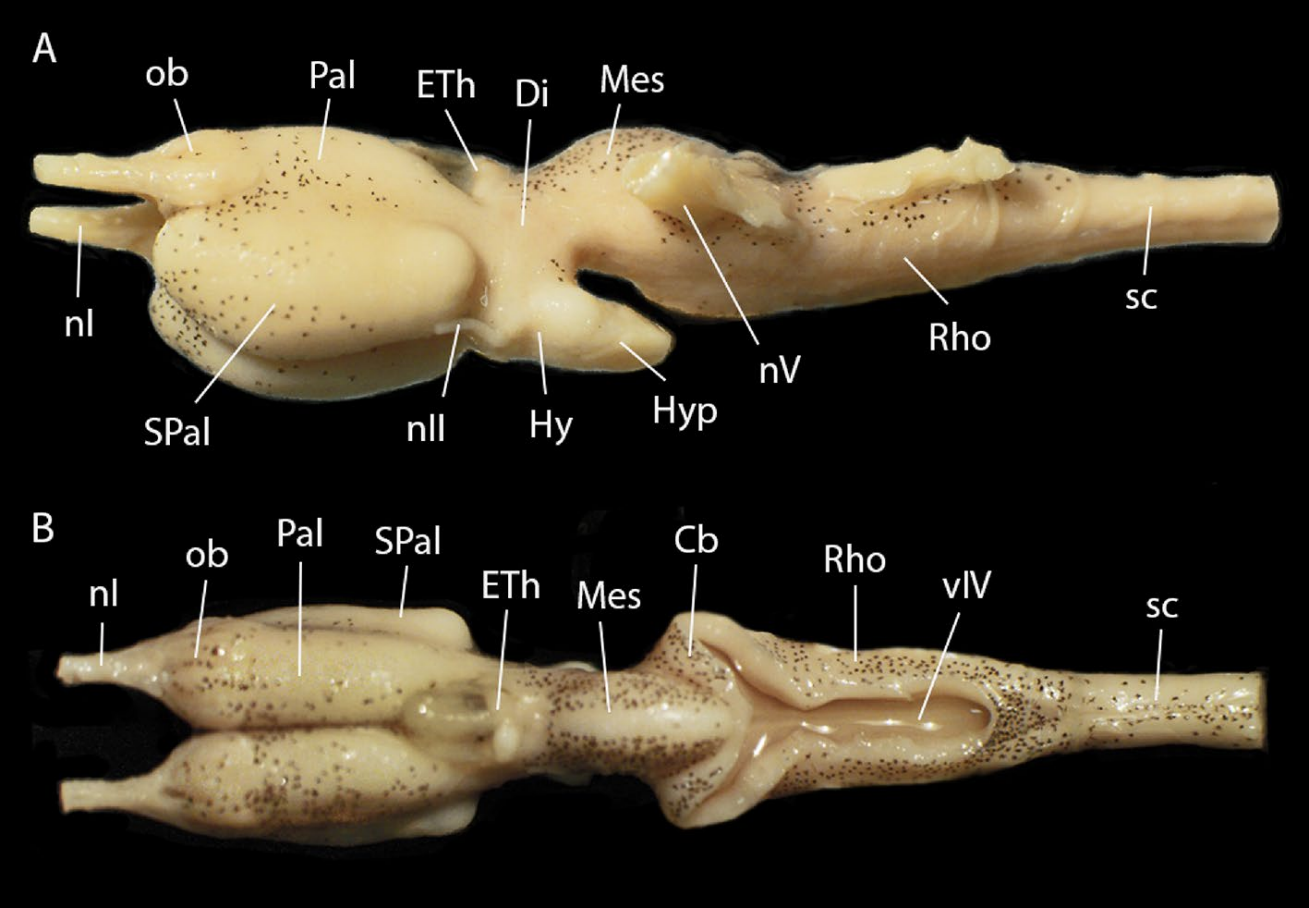
Brain of *Protopterus annectens* in lateral (**A**) and dorsal (**B**) views. *Cb* cerebellum, *Di* diencephalon, *ETh* epithalamus, *Hy* hypothalamus, *Hyp* hypophysis, *Mes* mesencephalon, *nI* olfactory nerve, *nII* optic nerve, *nV* trigeminal nerve, *ob* olfactory bulb, *Pal* pallium, *Rho* rhombencephalon, *sc* spinal cord, *SPal* subpallium, *vIV* fourth ventricle.

Within the sarcopterygians, lungfishes and coelacanths are the only extant groups of fishes and the closest living relatives of tetrapods^1–7^. Although in the past lungfishes were much more abundant, especially during the Devonian^8^, currently only six species survive, belonging to the genera *Lepidosiren* (one South American species), *Neoceratodus* (one Australian species), and *Protopterus* (four African species). The comparison between the living species and the fossil records reveals the persistence of ancestral traits due to the slow evolution experienced by these fishes^9,10^. Among the main features of these animals stands the presence of lungs, which are homologue to those of tetrapods and, consequently, allow them to breathe air^11,12^. Regarding its central nervous system (Fig. 1), one of the main characteristics is that the pallium develops by evagination in a similar way to tetrapods, which differentiates them from all actinopterygian fish, whose pallium is everted^13^. Thus, the phylogenetic position of lungfish is crucial for understanding the evolutionary processes of basal tetrapods. In neuroanatomical terms, the study of lungfish has aroused great interest since early anatomical studies from the late nineteenth century and throughout the twentieth century^14–19^, which, based on classical histological techniques, describe in detail the main characteristics of the brain of these vertebrates. It was not until more recently that a limited number of more advanced neuroanatomical histological studies have been published, not only from a general perspective^20–22^, but also describing different neurotransmission systems and the expression patterns of various transcription factors^23–37^. These studies have provided exceptional information that has made it possible to identify the different brain territories of these models as well as their cell types. However, these high-resolution techniques at the cellular level also have technical disadvantages such as the impossibility of maintaining the integrity of the tissue due to deformations caused by manipulation. This problem can be avoided by using non-destructive techniques, such as X-ray computer tomography^38^, synchrotron-radiation X-ray tomography^39^, optical projection tomography^40^, ultrasonography^41^, and magnetic resonance imaging, each technique with particular advantages and limitations^42^.

Among these methods to study neuroanatomy, magnetic resonance imaging (MRI) stands out as a non-invasive technology that allows tomography analysis and obtaining anatomical images, with advantages such as the high level of tissue contrast and the lack of ionizing radiation, and limitations due to the problems derived from a possible high magnetic field strength, like physiological effects or the generation of artifacts^42^. In addition, although all these techniques do not have the cellular resolution obtained with the post mortem histochemical analysis, they offer other advantages, such as the possibility of analyzing the tissue in vivo, as well as the availability of rare models in the laboratory, which may be accessible to the scientific community through the large number of resources that have been emerging in recent years with these analyses. In fact, apart from its main clinical use in humans, or even in non-humans, its use in research is becoming more widespread in the last years, as evidenced by the project Digital Fish Library for the case of fishes (http://www.digitalfishlibrary.org/). Recently, studies of the cranial endocast of sarcopterygian fishes have been developed using non-invasive technology, either with fossil or living species^43–45^. However, these approaches lack a detailed neuroanatomical interpretation. In this study, we have identified and segmented for the first time the main brain regions of a representative species of lungfish, *Protopterus annectens*, using MRI approach. Thus, we have developed a three-dimensional (3D) model of the brain showing its main regions and the ventricular system, as well as providing volumetric measures of the brain and the ventricular system.

## Results and discussion

**Figure 2:**
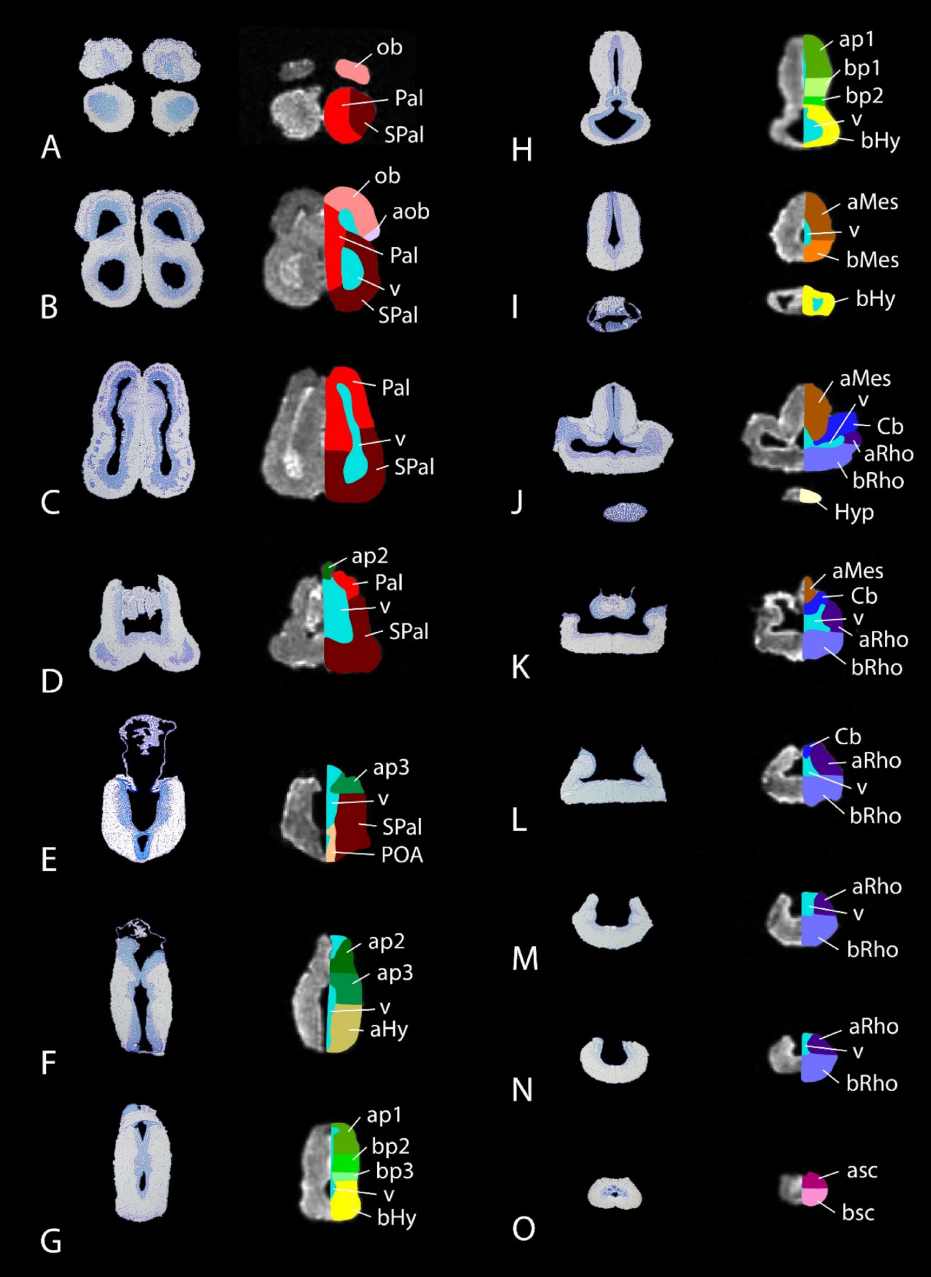
Coronal Nissl-stained and MRI sections of the brain of *Protopterus annectens*, including on the right side the segmentation of the different brain regions following the color scheme detailed in Table 1. *aHy* alar hypothalamus, *aMes* alar mesencephalon, *aob* accessory olfactory bulb, *ap1* alar part of p1, *ap2* alar part of p2, *ap3* alar part of p3, *aRho* alar rhombencephalon, *asc* alar spinal cord, *bHy* basal hypothalamus, *bMes* basal mesencephalon, *bp1* basal part of p1, *bp2* basal part of p2, *bp3* basal part of p3, *bRho* basal rhombencephalon, *bsc* basal spinal cord, *Cb* cerebellum, *Hyp* hypophysis, *ob* olfactory bulb, *Pal* pallium, *POA* preoptic area, *SPal* subpallium, *v* ventricle.

**Figure 3:**
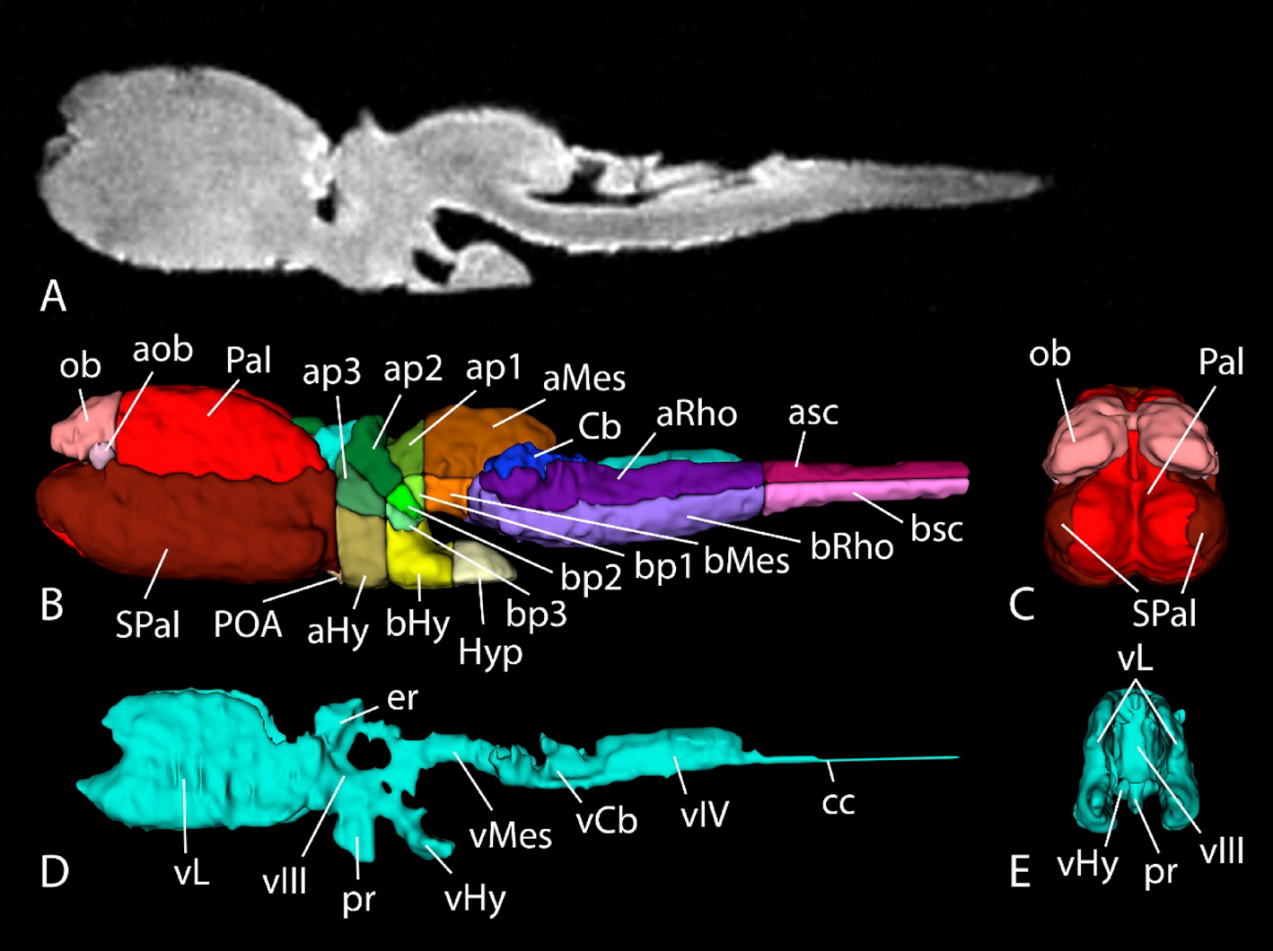
MR image (**A**) and 3D reconstructions of the brain (**B**,**C**) and the ventricular system (**D**,**E**) of *Protopterus annectens* in sagittal (**A**,**B**,**D**) and frontal (**C**,**E**) views. *aHy* alar hypothalamus, *aMes* alar mesencephalon, *aob* accessory olfactory bulb, *ap1* alar part of p1, *ap2* alar part of p2, *ap3* alar part of p3, *aRho* alar rhombencephalon, *asc* alar spinal cord, *bHy* basal hypothalamus, *bMes* basal mesencephalon, *bp1* basal part of p1, *bp2* basal part of p2, *bp3* basal part of p3, *bRho* basal rhombencephalon, *bsc* basal spinal cord, *Cb* cerebellum, *cc* central canal, *er* epithalamic recess, *Hyp* hypophysis, *ob* olfactory bulb, *Pal* pallium, *POA* preoptic area, *pr* preoptic recess, *SPal* subpallium, *vCb* cerebellar ventricle, *vHy* hypothalamic ventricle, *vIII* third ventricle, *vIV* fourth ventricle, *vL* lateral ventricles, *vMes* mesencephalic ventricle.

**Figure 4:**
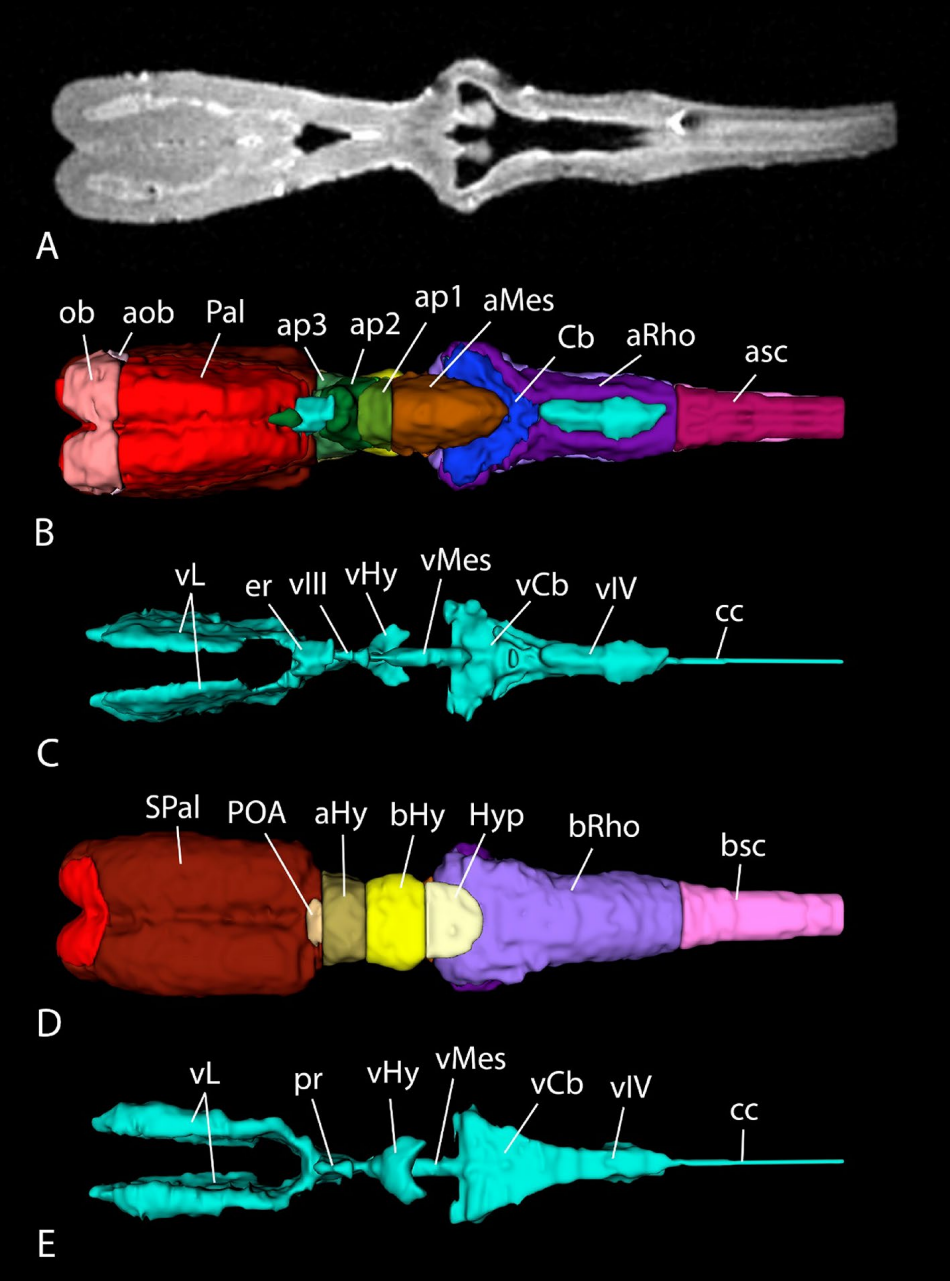
Horizontal MR image (**A**) and 3D reconstructions of the brain (**B**,**D**) and the ventricular system (**C**,**E**) of *Protopterus annectens* in dorsal (**B**,**C**) and ventral (**D**,**E**) views. *aHy* alar hypothalamus, *aMes* alar mesencephalon, *aob* accessory olfactory bulb, *ap1* alar part of p1, *ap2* alar part of p2, *ap3* alar part of p3, *aRho* alar rhombencephalon, *asc* alar spinal cord, *bHy* basal hypothalamus, *bRho* basal rhombencephalon, *bsc* basal spinal cord, *Cb* cerebellum, *cc* central canal, *er* epithalamic recess, *Hyp* hypophysis, *ob* olfactory bulb, *Pal* pallium, *POA* preoptic area, *pr* preoptic recess, *SPal* subpallium, *vCb* cerebellar ventricle, *vHy* hypothalamic ventricle, *vIII* third ventricle, *vIV* fourth ventricle, *vL* lateral ventricles, *vMes* mesencephalic ventricle.

**Figure 5:**
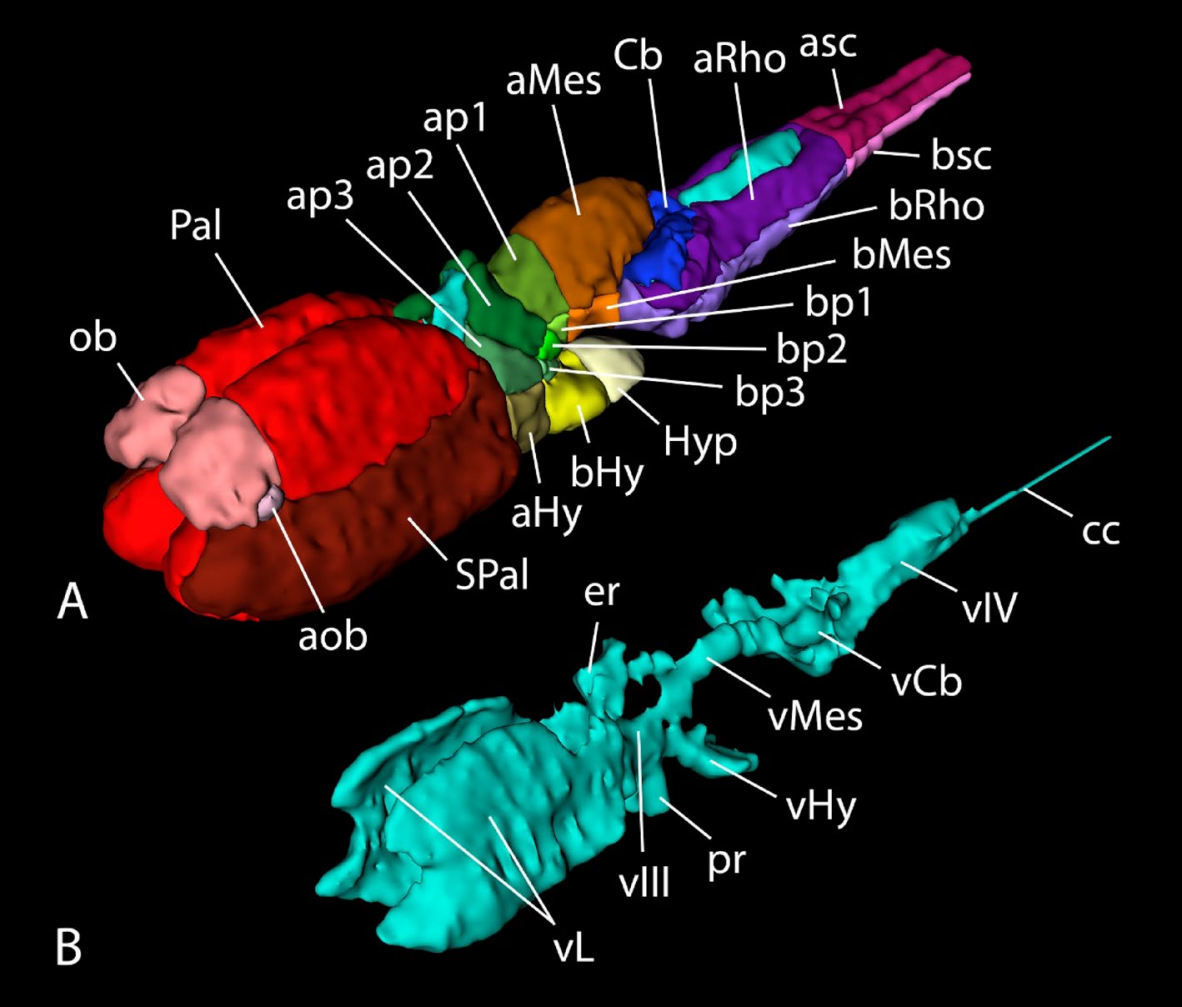
Oblique 3D reconstructions of the brain (**A**) and ventricular system (**B**) of *Protopterus annectens*. *aHy* alar hypothalamus, *aMes* alar mesencephalon, *aob* accessory olfactory bulb, *ap1* alar part of p1, *ap2* alar part of p2, *ap3* alar part of p3, *aRho* alar rhombencephalon, *asc* alar spinal cord, *bHy* basal hypothalamus, *bMes* basal mesencephalon, *bp1* basal part of p1, *bp2* basal part of p2, *bp3* basal part of p3, *bRho* basal rhombencephalon, *bsc* basal spinal cord, *Cb* cerebellum, *cc* central canal, *er* epithalamic recess, *Hyp* hypophysis, *ob* olfactory bulb, *Pal* pallium, *pr* preoptic recess, *SPal* subpallium, *vCb* cerebellar ventricle, *vHy* hypothalamic ventricle, *vIII* third ventricle, *vIV* fourth ventricle, *vL* lateral ventricles, *vMes* mesencephalic ventricle.

The brain of the lungfish *P. annectens* has been divided in the main regions shared by most vertebrates: olfactory bulb, telencephalon, hypothalamus, diencephalon, mesencephalon, cerebellum, and rhombencephalon. In addition, other subdivisions, such as the accessory olfactory bulb, pallium, subpallium, and preoptic area within the telencephalon, the hypophysis, and the alar-basal boundary, have also been identified and analyzed in this study. The segmentation of the different regions, together with the ventricular system, was performed manually using previous neuroanatomical studies as reference^23–28,31,35,36^ (Fig. 2; see list of colors and abbreviations in Table 1). Based on these segmentations, a three-dimensional model was built (Figs. 3, 4 and 5). The data of the variables considered (body length and weight, brain and ventricular volumes, and the relation between the latter two) are detailed in Table 2, while the correlation analyses of them appear in Table 3. The correlation observed between the variables body length, body weight and brain volume is high, as well as the correlation between brain and ventricular volumes. In the case of the latter, the correlation with the rest of the variables is considerable, but lower, most probably due to the sometimes-difficult interpretation of the extent of the ventricles by MRI, particularly in zones like the third or the fourth ventricle, where the *tela choroidea* is not clearly distinguishable with this technique.

**Table 1:**
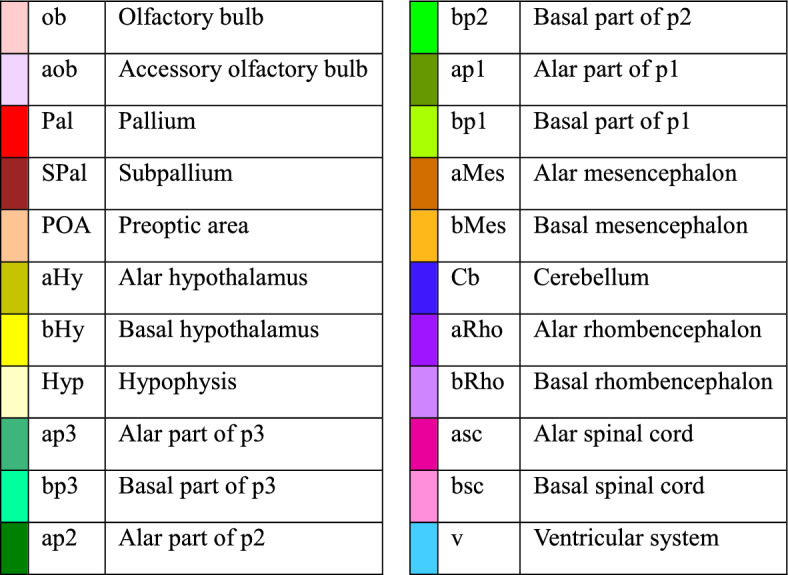
List of the segmented brain regions and associated colors and abbreviations.

**Table 2:**
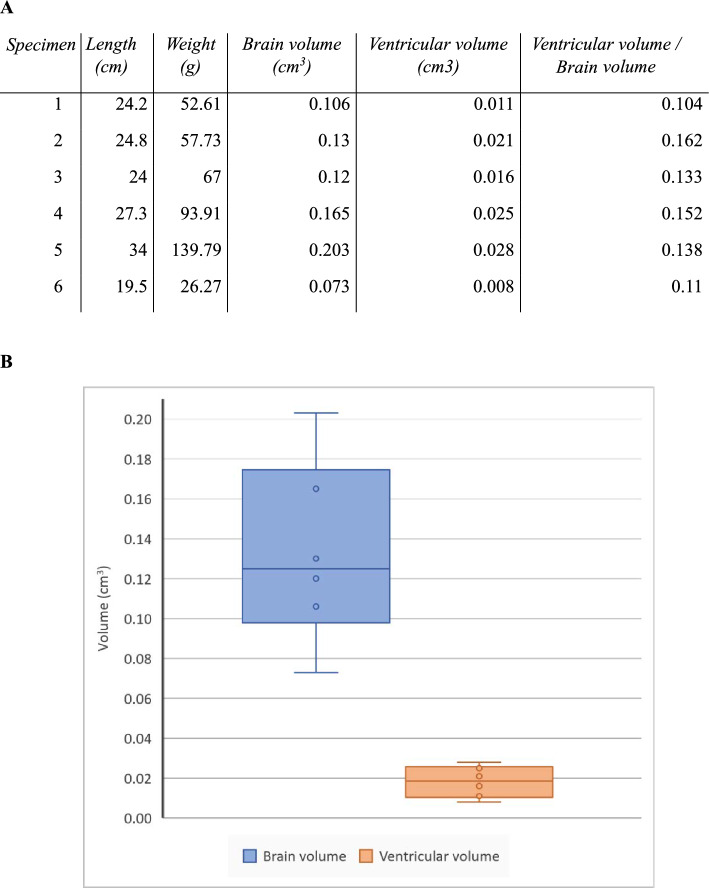
(A) Data of the variables measured and (B) chart of the variables brain and ventricular volumes.

**Table 3:** Correlation analysis of the variables length and weight of the brain, brain volume, and ventricular volume.

	Length	Weight	Brain volume	Ventricular volume
Length	1			
Weight	0.98271813	1		
Brain volume	0.97300469	0.98019393	1	
Ventricular volume	0.88264345	0.89499899	0.960870023	1

This neuroanatomical technique has been widely used in humans, mostly with a clinical purpose^46,47^, but, like in our case, it has also been leveraged to study the brains of several models of all groups of vertebrates, i.e. mammals^48,49^, birds^50–52^, reptiles^53–55^, amphibians^56^, and fish^57–62^. Lungfishes are a key model in evolutionary analysis due to their phylogenetic position, since they constitute the only bony fish with an evaginated brain more closely related to amphibians than to other fishes. Classical histological^18–20^ and recent genoarchitectonic studies^23–27^ have suggested that these neuroanatomical features may be comparable to those of the ancestors of amniotes. Therefore, the neuroanatomic characteristics that we are going to detail below could be discussed in this context.

### Olfactory bulb

Located in the anterodorsal telencephalon, and sessile on the rostral part of the telencephalic hemispheres, the secondarily evaginated olfactory bulbs of *Protopterus* present a laminar structure and large ventricles (Fig. 2A,B). In addition, in the ventrolateral part appears the accessory olfactory bulb in caudal levels^26^ (Figs. 3B, 5A). In particular, regarding the vomeronasal system, it was shown that, in terms of processing this information, lungfishes share a basic organization with tetrapods^26^, which allowed us to suggest that these features were already present in the last common ancestor of lungfishes and tetrapods.

### Pallium and subpallium

The telencephalic roof houses the pallium, while the subpallium (septum medially and striatum laterally) is located in the floor (Figs. 3B, 4B,D, 5A). Although there is no external evidence of the pallial-subpallial boundaries, internally they are revealed by changes in cellular density and thickness of the cell plate. However, the divisions of the different pallial fields, i.e., medial, dorsal, lateral and ventral, and the subpallial nuclei were not clearly distinguishable by MRI (Fig. 2A–E). The telencephalon of this model exhibits one of the unique characteristics of sarcopterygian fishes, which is its development by evagination, in contrast to actinopterygian fishes whose telencephalon develops by eversion. This feature, together with its neurochemistry and regionalization (the organization of the pallium into at least four domains^24^, the existence of subpallial regions comparable to that of tetrapod^29,33^, as well as the conserved neurochemical systems^24,34,36^), has made the telencephalon of this model very similar in appearance to that of amphibians, and in particular to anurans^27,37^. From an evolutionary point of view this is of great interest as it supports the possible proximity of these model to the ancestor of amniotes and its position as a key model in these evolutionary studies.

### Preoptic area

This caudal subpallial region develops from the nonevaginated part of the telencephalon^63^. It is situated ventrally, in the territory of the preoptic recess of the third ventricle (Figs. 2E, 4D), with which most of its cells send cerebrospinal fluid contacts. The neurochemistry, genoarchitecture and organization of this region has been shown to be highly conserved with respect to that described in tetrapod sarcopterygians^25,32,33^.

### Hypothalamus

Following the prosomeric model, the alar-basal boundary courses along the rostrocaudal extension of the brain, but folds almost 90º in the diencephalic region, dividing the hypothalamus in an alar portion (rostral in classical view, but dorsal in the updated prosomeric view) and a basal portion (caudal in classical view, but ventral in prosomeric view) (Figs. 3B, 4D, 5A). The alar portion houses the paraventricular (with the neurosecretory nuclei) and the subparaventricular regions, the latter of which contains the suprachiasmatic nucleus (Fig. 2F), while the basal portion is composed of the tuberal and the mamillary areas (Fig. 2G–I). The interpretation of the hypothalamus of this model following the subdivisions proposed in the prosomeric model is recent, and it has been based on the expression of main morphogens of this basal territory such as Nkx2.1 and Isl1^33^ and the neurochemistry of the hypothalamus itself, as reflected in the catecholaminergic populations^31^, which are highly conserved in evolution. In addition, also in the caudal part of the basal portion is situated the prominent hypophysis (Figs. 2J, 3B, 4D, 5A).

### Diencephalon

This region can be segmented in 3 prosomeres that possess alar and basal portions: p3, which includes the prethalamic eminence and prethalamus in its alar portion and the posterior tubercle in the basal part; p2, whose alar portion is composed of the thalamus and the epithalamus, including the habenula and the epiphysis; and p1, with the pretectum in its alar part, whereas the basal portions of p1 and p2 are smaller (Figs. 2F–H, 3B, 4B, 5A). These MRI-evidenced subdivisions have been precisely delineated on the basis of the expression of Pax6 transcription factors in combination with Isl1 expressed in the prethalamus^36^, as well as its boundary with the calcium-binding protein-rich thalamus^34^. The combination of both types of approaches has allowed the interpretation of MRI studies to have much greater depth of analysis based on this prior information.

### Mesencephalon

It is segmented in an alar portion, which houses the optic tectum and the torus semicircularis, and the basal portion, where the mesencephalic tegmentum is situated housing the oculomotor nucleus (Figs. 2I–K, 3B, 4B, 5A). These MRI results in the case of the midbrain are particularly interesting because they allow us to demonstrate its relative size, already described but without exact measurements, since the midbrain of lungfish represents a very small portion of the brain, with a bilaterally poorly developed optic tectum^18,19^, which may be a reflection of their ecological adaptations^22^. Although neurochemical populations conserved in other vertebrates have been described, as well as evolutionary particularities specific to lungfish, such as the superficial mesencephalic nucleus, located in a subpial position in the lateroventral margin of the alar midbrain^27^, the definition in this case is scarce.

### Rhombencephalon

It is the most caudal region of the brain, divided into rhombomeres containing alar and basal portions (Figs. 2J–N, 3B, 4B,D, 5A). There have been described eight rhombomeres, plus r0 or the isthmic region in the most rostral part, which houses the trochlear nucleus. All the motor nuclei of the cranial nerves, except for the oculomotor nucleus, are situated in the basal rhombencephalon. The basal portion also houses the reticular formation, with important components like the serotonergic raphe column in the medial line. In turn, the alar portion harbors the noradrenergic locus coeruleus, the cholinergic laterodorsal tegmental nucleus, the acoustic-vestibular nuclei and the nucleus of the solitary tract. Caudal to the obex, in the most caudal part of the rhombencephalon, begins the rostral portion of the spinal cord (Figs. 2O, 3B, 4B,D, 5A).

### Cerebellum

Within the rhombencephalon and poorly developed, this region expands from the alar part of r1 and folds caudally over the rostral fourth ventricle (Figs. 2J–L, 3B, 4B, 5A). In this region, which had already been defined since classical studies^14–16^ and more recently on the basis of Pax6 expression in its granule cells^36^, again, MRI analysis is very useful to identify it, as it is clearly observed and can be confidently defined rostrocaudally. It also opens the door to volumetric comparative studies.

### Ventricular system and volumetric analysis

The MRI technology makes it possible to analyze accurately the ventricular system in a way that is unfeasible using histological techniques, that is, keeping it unaltered by possible deformations or contractions that are common when manipulating the tissue. Thus, we were able to develop a 3D representation of the entire system of cavities that greatly improves the understanding of its organization. In the telencephalon, two large lateral ventricles comprise its dorsoventral extent, from the pallium to the subpallium, reaching rostrally the olfactory bulbs and caudally joining into one cavity at levels corresponding to the caudal extent of the pallium (Figs. 2B–D, 3D,E, 4C,E, 5B). This union results in the third ventricle (Figs. 2E–H, 3D,E, 5B), which occupies the diencephalon. From its ventral part, the preoptic recess develops rostrally (Figs. 2E, 3D, 4E) and the hypothalamic ventricle caudally (Figs. 2G,H, 3D,E, 4C,E, 5B), the latter forming two lateral recesses in its caudalmost part (Fig. 2I). In turn, in its dorsal part stands the epithalamic recess between the epiphysis and the habenulae (Fig. 2E,F). The third ventricle is then succeeded by the mesencephalic ventricle (Figs. 2I, 3D, 4C,E, 5B), and this one leads caudally to the cerebellar ventricle, which forms two lateral extensions in its rostral part (Figs. 2J,K, 3D, 4C,E, 5B). Finally, the rest of the rhombencephalon is occupied by the fourth ventricle (Figs. 2L-N, 3D, 4B,C,E, 5B), which is opened dorsally and caudally closes itself to form the central canal that runs through the center of the spinal cord (Figs. 2O, 3D, 4C,E, 5B).

Therefore, MRI analysis of the ventricles has been of great interest and help, as it has allowed us to define these structures in lungfish with great precision for the first time. In addition, we have been able to perform a comparative volumetric study of these structures in relation to brain size (Tables 2 and 3). In relative size, the lungfish brain is large; however, the evolutionary interpretation of the cognitive consequences of having a large brain is not always obvious. Some authors have related the increase in cognitive capacities to an increase in the number of neurons, independently of volume^64^, while others consider that volume is an appropriate parameter since it is determined by the number of neurons^56^. In any case, regardless of the relationship with cognitive capacity, we consider that volumetric analyses by MRI are of great interest from an evolutionary point of view, not only in terms of total volume, but also in a partial way, between areas and specific territories. As discussed, in the case of lungfish, it would be very interesting to relate the relative size of the optic tectum or cerebellum to that of other models with different ecological characteristics and phylogenetic positions.

## Conclusions

The MRI analysis of the brain of the lungfish *Protopterus annectens* provides valuable information, unattainable by histological methods. On the one hand, it has enabled us to measure brain and ventricular volumes, data that can be contrasted with those measures in other vertebrates in order to develop comparative neuroanatomical studies. On the other hand, we were able to use the MRI data to develop the first 3D brain atlas of a lungfish, granting a better comprehension of the neuroanatomy of these sarcopterygian fishes.

## Materials and methods

### Animals

In this study seven young adult specimens of *Protopterus annectens* (total length 19.5–34 cm) were used. All the experiments described here were performed according to the ARRIVE guidelines and the regulations and laws established by the European Union (2010/63/EU) and Spain (Royal Decree 118/2021) for handling and care of research animals, and after acceptance of the Complutense University to conduct this research (ES-28079-0000086).

### Animal processing

All animals were profoundly anesthetized by immersion in 0.2% tricaine methanesulfonate solution (MS222, pH 7.4; Sigma-Aldrich Merck KGaA, Darmstadt, Germany) and perfused transcardially with 200 ml of 4% paraformaldehyde in 0.1 M phosphate buffer (PB, pH 7.4). The brain and the rostral spinal cord were extracted and stored firstly in the same fixative solution for 3 h at 4 °C and then in a solution of 30% sucrose in PB overnight at 4 °C.

### MRI procedure

MRI studies were performed at BioImaC (ICTS BioImagen Complutense, Madrid, Spain), node of the ICTS ReDIB (https://www.redib.net/). Six of the brains were dedicated for the MRI analysis. They were drained and immersed in a proton-free susceptibility-matching fluid (Fluorinert^®^ FC-40; Sigma-Aldrich, Saint Louis, MO, USA) and introduced in the MRI scanner (Icon 1 T-MRI; Bruker BioSpin MRI GmbH, Ettlingen, Germany). The system consists of a 1 Tesla permanent magnet with a gradient coil that provides a gradient strength of 450 mT/m and a solenoid RF coil. Three-dimensional T2 coronal weighted images were acquired by using a rapid acquisition with relaxation enhancement (RARE) technique, with predefined parameters (repetition time = 2000s; echo train length = 8; interecho interval = 27 ms; effective echo time = 90 ms; number of averages = 18; field of view = 26 × 7 × 7 mm^3^). The acquired matrix size was 260 × 70 × 70 (resolution 0.1 × 0.1 × 0.1 mm) and the total acquisition time around 90 min. All MRI data were analyzed with ImageJ software (version 1.54f, Wayne Rasband, NIH, USA; http://imagej.org). Brain areas identification and segmentation were performed manually, and the volumes of the brain and the ventricular system were measured. The three-dimensional model was assembled with 3D Slicer software (version 1.6; https://www.slicer.org/).

### Histological procedure

The other brain was processed for Nissl staining. In this way, it was introduced in a solution of 20% gelatin with 30% sucrose in PB, resulting in a block that was stored overnight in a 3.7% formaldehyde solution at 4 °C. The block was cut on a freezing microtome in 40 µm thick transverse slices, which were collected and rinsed in cold PB. The sections were then mounted on glass slides and stained with cresyl violet, and finally covered with Entellan^®^ (Sigma-Aldrich Merck KGaA). Photographs were taken with an Olympus DP74 camera coupled on an Olympus BX51 microscope (Olympus, Tokyo, Japan) and adjusted with Photoshop (Adobe Systems, San Jose, CA).

The nomenclature used in the present study is the same that in previous studies of the brain of lungfish^25,26,28–37^.

## Data Availability

The raw data supporting the conclusions of this study are available on Zenodo (doi: 10.5281/zenodo.10676292).
